# Comparison of Psychosocial Distress in Areas With Different COVID-19 Prevalence in Korea

**DOI:** 10.3389/fpsyt.2020.593105

**Published:** 2020-11-24

**Authors:** Mina Kim, In-Hoo Park, Young-Shin Kang, Honey Kim, Min Jhon, Ju-Wan Kim, Seunghyong Ryu, Ju-Yeon Lee, Jae-Min Kim, Jonghun Lee, Sung-Wan Kim

**Affiliations:** ^1^Gwangju Mental Health and Welfare Commission, Gwangju, South Korea; ^2^Department of Nursing, Graduate School, Chonnam National University, Gwangju, South Korea; ^3^Department of Psychology, Chonnam National University, Gwangju, South Korea; ^4^Department of Psychiatry, Chonnam National University Medical School, Gwangju, South Korea; ^5^Daegu Metropolitan Mental Health and Welfare Center, Daegu, South Korea; ^6^Department of Psychiatry, Catholic University of Daegu, College of Medicine, Daegu, South Korea

**Keywords:** COVID-19, prevalence, pandemic, psychosocial stress, anger, activity

## Abstract

**Objectives:** This study aimed to compare psychosocial distress in areas in Korea with different prevalence of coronavirus disease 2019 (COVID-19) after validating a questionnaire on psychological experiences and stress associated with the disease outbreak.

**Methods:** Using an online-based survey, psychosocial distress associated with COVID-19 was investigated in three regions, which were selected according to their prevalence of COVID-19. A total of 1,500 people from an online public panel in the three regions participated in the study. The questionnaire included sociodemographic information, psychosocial experience and stress related to COVID-19, and the perceived stress scale (PSS), patient health questionnaire-9 (PHQ-9), and generalized anxiety disorder-7 (GAD-7). Those questionnaires and scales were compared by level of prevalence of COVID-19 after validating the questionnaire on psychosocial distress associated with COVID-19.

**Results:** The 19 items on psychosocial experience associated with COVID-19 comprised 5 subscales, with favorable Cronbach's α ranging from 0.69 to 0.88. Six stress items related to COVID-19 had a Cronbach's α of 0.79. Disturbance in eating and sleeping, difficulty with outside activities, stress from COVID-19, and PSS scores were greater in the areas where COVID-19 was highly prevalent. Economic problems, daily activity changes, and anger toward society were higher in the higher-prevalence regions.

**Discussion:** Psychosocial distress associated with COVID-19 was closely related to the prevalence of the disease in the areas where participants lived. Psychosocial interventions for distress associated with COVID-19 should be developed and prepared for people during this lengthy pandemic.

## Introduction

Coronavirus disease 2019 (COVID-19) has been rampant around the world since the World Health Organization (WHO) declared a pandemic on March 11, 2020 (1). As of June 19, 2020, COVID-19 has occurred in 216 countries, resulting in 8,242,999 infected and 445,535 dead (2). This novel viral infection affects all populations and all age groups (3), and there is no vaccine available. WHO and national quarantine authorities are focusing on preventing transmission through social distancing, quarantine, and self-isolation (4). External activities of individuals are limited, and stress is exacerbated by difficulties in buying necessary goods and overload of information from media (5). An epidemic of infectious disease affects psychological as well as physical health. Recent studies have shown that the general public has had lower psychological well-being and higher anxiety and depression scores during the pandemic than before the occurrence of COVID-19 (6). Even if the spread of COVID-19 stops, there will be a need for measures against psychosocial sequelae.

In Korea, the first patient was confirmed on January 20, 2020. Since then, the Korea Centers for Disease Control & Prevention has focused on delaying the inflow of viruses and the spread of the disease in the community, measures that have achieved some effects. However, after February 18, 2020, when the 31st patient was found in Daegu city, the situation began to change rapidly with collective infection and nationwide spread (7). At the start of the present study (April 22, 2020), there were 10,694 confirmed COVID-19 cases, including 238 deaths, in Korea. Daegu city had 6,836 confirmed cases, and the incidence rate per 100,000 population was 281.1. This accounted for 63.9% of the occurrence in Korea and was its highest rate. There were 1,286 confirmed cases in the Capital area, including Seoul metropolitan city and Gyeonggi-do province, accounting for 12% of all infected, with an incidence rate of 5.6 per 100,000 population. This area has a population of 23,038,041, which is 44.3% of the national population, and has the highest population density. Gwangju, which has a population density similar to that of Daegu, had 30 confirmed cases and an incidence rate per 100,000 population of 2.1, which was the lowest level of infection in Korea (Table 1) (8, 9).

**Table 1:** Present condition of epidemic areas.

**Areas**	**Population**	Population density^*^	**COVID-19**^**†**^		
			**Confirmed cases**	**Deaths**	**Incidence**
	***N* (%)**	**Population/km^**2**^**	***N* (%)**	***N* (%)**	**Per 100,000**
South Korea	51,842,524 (100.0)	509.2	10,694 (100.0)	238 (100.0)	20.6
Daegu	2,431,523 (4.7)	2,791.0	6,836 (63.9)	165 (69.3)	281.1
Capital area	23,038,041 (44.4)	16,364.0^‡^	1,286 (12.0)	16 (6.7)	5.6
Gwangju	1,456,096 (2.8)	2,998.8	30 (0.3)	0 (0.0)	2.1

* As of 2015;

† As of April 22, 2020;

‡ *Seoul*.

COVID-19, which is characterized by high propagation power, is estimated to have different psychosocial effects according to the prevalence in an area and its population density. This study aimed to grasp the characteristics of psychosocial behavioral changes and distress according to the level of the epidemic, and validate a questionnaire to investigate psychosocial stress and burden. The results of this study can be used as evidence for a psychosocial management strategy for the COVID-19 pandemic and for further study to investigate its impact.

## Methods

### Study Design and Participants

The present study is a part of mental health survey on the psychosocial effects of COVID-19 in the general population and patients with mental problems. This study analyzed data from the general population to investigate the general influence of COVID-19 in areas with different prevalence of the disease, while identifying a reliable questionnaire for psychosocial distress associated with COVID-19. An online survey of the general population was conducted. Three geographic areas differing in COVID-19 prevalence (low, intermediate and high) were surveyed, and participants were recruited using the quota sampling method, with consideration of age and gender. The inclusion criteria were an age of 19–65 years and residing in one of the three study regions. The gender and age distributions were identical among regions. There were 500 participants each in Daegu, Capital area (Seoul/Gyeonggi-do), and Gwangju. The survey was conducted through an online survey service provider (Macromill Embrain), which has 1,324,315 people available to take part in surveys, all of whom have an individual identification number. Participants can only take part after providing consent for the use of personal information. For this survey, an invitation email was sent to 4,065 people in the study regions, and participation was voluntary. After clicking on the link to the survey page, the informed consent form was presented. Following provision of consent, the participants indicated their age and area of residence; if they met the selection criteria, the survey began. Survey responses could not be reviewed or changed using the browser's back button. When a participant clicked the submit button on the final page, the survey was considered complete and could not be repeated. A total of 1,819 people completed the questionnaire; 319 were excluded from the final analysis (because they responded with the same answer option throughout, or very quickly, etc.), such that we ultimately had data for 1,500 respondents. The data collection period was from April 24 to May 5, 2020. The study was approved by the Chonnam National University Hospital Institutional Review Board (CNUH-2020-092). Electronic informed consent was obtained from each participant prior to starting the investigation.

### Measures

#### Socio-Demographic Information

Socio-demographic data were collected on gender, age, residential location, marital status, religion, education level, medical security, employment status, smoking status, and drug use related to physical and mental health.

#### Psychosocial Experience and Stress Associated With COVID-19

To identify characteristics of psychosocial behavioral changes in the subjects following the onset of the COVID-19 epidemic, a questionnaire was developed by the authors based on our own clinical experiences and evidence and the existing literature on other infectious disease epidemics and stress. Twenty-one questions were prepared to measure the psychosocial experiences associated with the COVID-19 epidemic, coupled with six questions to measure stress associated with COVID-19. The 21 psychosocial experience questions consisted of 10 questions based on the existing literature (10–12) and 11 self-elaborated questions. Two questions on fear of infection (10), five questions on changes in daily life (12), and three questions on stigma (11) were based on the literature but with modifications. The 21 questions of psychosocial experience and 6 questions related to COVID-19 stress were identified through item analysis and exploratory factor analysis (Tables 2, 3). All items in the questionnaires were rated using a 5-point Likert scale (1, strongly disagree; 2, disagree; 3, neutral; 4, agree; 5, strongly agree). The scale was developed in Korean.

**Table 2:** Questionnaire for COVID-19-related psychosocial distress item analysis and exploratory factor analysis.

**No**	Item contents^*^	**Item analysis (*****n*** **=** **1,500)**							**EFA (*****n*** **=** **1500)**				
		**M**	**SD**	**ITC**	**Alpha if item deleted**	**Comm-unality**	**Skew-ness**	**Kurtosis**	**Factor loading**				
									**1**	**2**	**3**	**4**	**5**
**Fear of COVID-19 infection**													
10	I am afraid I will get COVID-19.	3.78	1.01	0.73	0.86	0.72	−0.68	−0.03	**0.81**	0.16	0.10	0.11	0.14
11	I am afraid my family will get COVID-19.	4.12	0.90	0.67	0.86	0.69	−1.06	1.08	**0.80**	0.20	0.02	0.03	0.11
12	I am afraid my family or I will die from COVID-19.	3.53	1.19	0.68	0.86	0.67	−0.43	−0.78	**0.78**	0.04	0.12	0.20	0.03
13	I am afraid there will be asymptomatic infected people around me.	3.77	0.98	0.71	0.86	0.62	−0.68	0.05	**0.73**	0.15	0.15	0.14	0.18
18	I am afraid my health will deteriorate due to restrictions on hospital visits.	3.34	1.11	0.69	0.86	0.58	−0.29	−0.73	**0.63**	0.02	0.33	0.16	0.23
14	I am afraid I will be quarantined for 2 weeks on contact with an infected person.	3.71	1.10	0.68	0.86	0.59	−0.69	−0.24	**0.61**	0.09	0.07	0.16	0.43
17	I am afraid when I am infected that I will infect others around me.	4.39	0.77	0.51	0.87	0.54	−1.45	2.60	**0.55**	0.33	−0.16	−0.10	0.30
**Difficulty in outside activities**													
4	COVID-19 is disrupting my personal itinerary and planning (travel, vacation, and so forth).	4.36	0.83	0.45	0.87	0.61	−1.50	2.44	0.15	**0.76**	0.06	0.01	0.09
2	Because of the fear of infection, I am limited to where I can go when I go out (eating out, movies, shopping, and so forth).	4.41	0.74	0.49	0.87	0.60	−1.51	3.35	0.27	**0.72**	0.09	0.04	−0.01
5	COVID-19 is disrupting official schedules and plans (business trips, workshops, exams, classes, and so forth).	4.12	0.99	0.39	0.87	0.53	−1.19	1.08	−0.02	**0.69**	0.19	0.02	0.13
1	I am reluctant to use public transportation to and from school and commuting.	4.04	0.92	0.57	0.86	0.45	−0.93	0.64	0.42	**0.48**	0.17	0.11	0.01
**Disturbance in eating and sleeping**													
6	My diet has become irregular since the COVID-19 outbreak.	2.55	1.13	0.50	0.87	0.77	0.38	−0.65	0.15	0.09	**0.86**	0.04	0.03
8	My sleep schedule has become irregular since the COVID-19 outbreak.	2.72	1.19	0.48	0.87	0.72	0.20	−0.96	0.15	0.08	**0.83**	0.06	0.00
7	I eat instant food more often since the COVID-19 outbreak.	3.17	1.13	0.46	0.87	0.51	−0.22	−0.81	0.03	0.29	**0.63**	0.08	0.16
**Stigma of COVID-19**													
19	People infected with COVID-19 do harm to society.	3.29	1.07	0.42	0.87	0.72	−0.34	−0.59	0.09	0.11	−0.01	**0.84**	0.07
21	People infected with COVID-19 are abhorrent.	2.17	0.96	0.42	0.87	0.67	0.62	−0.12	0.07	−0.12	0.21	**0.74**	0.24
20	A person infected with COVID-19 has a fatal virus in his/her body.	3.13	1.10	0.46	0.87	0.64	−0.15	−0.76	0.29	0.10	0.02	**0.73**	−0.11
**Fear of blame for COVID-19 infection**													
15	I am afraid all of my movements (places/people visited) will be revealed when I am confirmed.	3.35	1.26	0.55	0.87	0.75	−0.28	−1.02	0.22	0.05	0.17	0.06	**0.82**
16	I am afraid I will be blamed by others when I am infected.	3.79	1.07	0.64	0.86	0.72	−0.70	−0.27	0.41	0.20	0.00	0.11	**0.70**
	Explained variance (%)								21.72	11.54	11.36	10.35	**8.84**
	Accumulative variance (%)								21.72	33.26	44.62	54.97	**63.81**
	Number of items								7	4	3	3	2
	Kaiser-Meyer-Olkin (KMO)										0.89		
	Bartlett's test of sphericity									*χ^2^* = 10,654.13	*df* = 171	*p < 0.001*	
	Reliability: Cronbach's α						Total = 0.87		0.88	0.75	0.69	0.75	0.69

* *All items on the questionnaires were rated using a 5-point Likert scale (1, strongly disagree; 2, disagree; 3, neutral; 4, agree; 5, strongly agree). M, Mean; SD, Standardized deviation; ITC, corrected item to total correlation; EFA, exploratory factor analysis. The highest item-factor-loading values are in bold*.

**Table 3:** Item analysis of stress questions related to COVID-19.

**Item No**	Item contents^*^	**Item analysis**							
		**M**	**SD**	**ITC**	**Alpha if item deleted**	**Comm-unality**	**Skewness**	**Kurtosis**	**Factor loading**
1	I experience distress from difficulties in obtaining masks or hand sanitizers	2.96	1.16	0.71	0.75	0.50	−0.03	−0.91	0.70
2	I have difficulties in obtaining daily necessities such as food or toilet paper	2.24	1.05	0.72	0.75	0.54	0.60	−0.30	0.73
3	I am experiencing economic stress (increased economic burden due to less income or more inflation)	3.51	1.15	0.69	0.76	0.46	−0.50	−0.56	0.68
4	I am distressed due to family responsibilities (increased family care or parenting burden)	2.95	1.20	0.73	0.75	0.53	−0.03	−0.94	0.73
5	I am distressed due to changes in daily activities (canceling appointments with friends, reducing external activities, and so forth)	3.69	1.02	0.62	0.77	0.37	−0.69	−0.03	0.61
6	I experience feelings of anger/resentment toward others or society	2.90	1.09	0.71	0.75	0.52	0.02	−0.73	0.72
	Explained variance (%)	49.14							
	Reliability: Cronbach's α	0.79							

* *All items on the questionnaires were rated using a 5-point Likert scale (1, strongly disagree; 2, disagree; 3, neutral; 4, agree; 5, strongly agree). M, mean; SD, standardized deviation; ITC, corrected item to total correlation*.

#### Psychological Characteristics

Existing scales were used to identify psychological characteristics. Stress was measured using the perceived stress scale (PSS) (13, 14), depression was measured using the Patient Health Questionnaire-9 (PHQ-9) (15, 16), and anxiety was measured using the generalized anxiety disorder-7 (GAD-7) scale (17).

### Statistical Analysis

First, the questionnaire about psychosocial experiences and distress was validated with item analysis, exploratory factor analysis, and reliability tests. Normal distribution was checked using the mean, standard deviation, skewness, and kurtosis of each item, and items with a correlation coefficient of 0.3 or more were selected (18). To confirm the suitability of the factor analysis, the Kaiser-Meyer-Olkin (KMO) test and Bartlett's test for sphericity were performed. The exploratory factor analysis was based on the principal components analysis of Varimax rotation, and items with a common variance or factor loading of 0.4 or more were selected (19). Reliability was verified by calculating Cronbach's α coefficient, which reflects internal consistency.

Next, we compared the scores by region using the chi-square test for categorical variables and one-way ANOVA for continuous variables. All scores in subfactors were represented as averages by dividing total scores by the number of items, ranging from 0 to 5. If the differences among groups were significant, *post-hoc* testing was conducted using a Scheffé test. The data collected in this study were analyzed using IBM SPSS Statistics 25.0 (IBM SPSS Statistics, New York, United States). All tests were two-tailed, with a significance level of *p* < 0.05.

## Results

### Item Analysis and Exploratory Factor Analysis

Analysis of 21 items related to psychosocial experience during the COVID-19 epidemic showed that the average score range of questions was 2.2 to 4.4 points, and the standard deviation range was 0.7 to 1.2 points. Skewness ranged from −1.51 to 0.62, and kurtosis ranged from −1.02 to 3.35. In the correlation analysis between each item and all items, the corrected item–total correlation was 0.4–0.7, and all items were higher than 0.3. The construct validity was confirmed through exploratory factor analysis. The KMO test was performed to determine whether the sample was suitable for exploratory factor analysis; it was found to be as high as 0.90, and Batlette's sphericity test showed that the correlation coefficient matrix was suitable for factor analysis (χ^2^ = 11,278.95, *p* < 0.001). After the two items (items A3, A9) with a communality or factor loading of 0.4 or less were deleted, a factor analysis was performed on 19 items: the KMO was as high as 0.89, and Batlette's sphericity test was also suitable (χ^2^ = 10,654.13, *p* < 0.001). As a result of exploratory factor analysis by performing orthogonal rotation Varimax using principal component analysis, subfactor was classified into five factors, and the cumulative explanatory amount was 63.8%. The first factor, “fear of COVID-19 infection,” was examined in seven questions, and the second factor was related to four questions associated with “difficulty in outside activities.” The third factor was “disturbance in eating and sleeping” with three questions, and the fourth factor was “stigma of COVID-19” with three questions. The fifth factor consisted of two questions and was named “fear of blame with COVID-19.” The reliability of each sub-factor was in the range of 0.69–0.88, and the total Cronbach's α was 0.87 (Table 2).

The stress associated with COVID-19 was probed with six questions; analysis showed that the average score range of the questions was 2.2 to 3.7 points, and the standard deviation range was 1.1 to 1.2 points. In the correlation analysis between each item and all items, the corrected item–total correlation was 0.6–0.7, and it was found to be one factor. Cronbach's α was 0.79 (Table 3).

### Characteristics of the Study Population

This study analyzed general population data for 1,500 people living in regions with high prevalence (Daegu), intermediate prevalence (Capital area), or low prevalence (Gwangju) of COVID-19. The proportion of female participants in each region was 50% (*n* = 250). The mean age of the participants was 40.2 ± 11.9 years, and did not differ by region. Marital status, education, employment status, medical insurance, religion, smoking status, and treatment of underlying physical and mental illness did not differ significantly among the regions (Table 4). People with experience of quarantine and COVID-19 tests of themselves and their family and acquaintances were significantly more frequent in the region with high prevalence (all *p*-values < 0.001).

**Table 4:** Characteristics of the study population in areas with different COVID-19 prevalence.

**Characteristics**	**Categories**	**Incidence of COVID-19**					
		**Total (*n* = 1,500)**	**High^**a**^ (*n* = 500)**	**Intermediate^**b**^ (*n* = 500)**	**Low^**c**^ (*n* = 500)**		
		***N* (%) or M ± SD**	***N* (%) or M ± SD**	***N* (%) or M ± SD**	***N* (%) or M ± SD**	***χ^2^ or F***	***P-value***
Age(years)		40.2 ± 11.9	40.5 ± 11.9	40.1 ± 11.9	40.2 ± 11.9	0.15	0.862
Marital status	Married	740 (49.3)	239 (47.8)	243 (48.6)	258 (51.6)	1.61	0.448
Education	≤ 12 years	256 (17.1)	79 (15.8)	86 (17.2)	91 (18.2)	1.03	0.598
Employment status	Employed	1,039 (69.3)	351 (70.2)	345 (69.0)	343 (68.6)	0.33	0.850
Medical insurance	Medicare	1,431 (95.4)	471 (94.2)	479 (95.8)	481 (96.2)	2.55	0.279
Religion	Yes	584 (38.9)	203 (40.6)	203 (40.6)	178 (35.6)	3.51	0.173
Smoking status	Smoker	320 (21.3)	99 (19.8)	114 (22.8)	107 (21.4)	1.34	0.511
Medication (physical health)	Yes	404 (26.9)	138 (27.6)	123 (24.6)	143 (28.6)	2.20	0.333
Medication (mental health)	Yes	71 (4.7)	23 (4.6)	25 (5.0)	23 (4.6)	0.12	0.943
Self-isolation	Yes	41 (2.7)	28 (5.6)	6 (1.2)	7 (1.4)	23.22	<0.001
COVID-19 test	Yes	54 (3.6)	34 (6.8)	6 (1.2)	14 (2.8)	23.97	<0.001
Positive (test result)	Yes	8 (0.5)	7 (1.4)	0 (0.0)	1 (0.2)	2.59	0.273
Quarantine or diagnosis (family or acquaintance)^*^	Yes	108 (7.2)	66 (13.2)	28 (5.6)	14 (2.8)	43.34	<0.001

a Daegu,

b Capital area,

c Gwangju,

* *Self-isolators or inspectors excluded*.

### Comparison of Psychosocial Behavioral Changes and Stress Associated With COVID-19

Table 5 shows the comparison of psychosocial behavioral changes and stress associated with COVID-19 according to region. Scores related to difficulty in outside activities were significantly lower in people in the region with low prevalence than in the other two regions (*F* = 12.36, *p* < 0.001). Scores on the disturbance in eating and sleeping factor were significantly greater in people in the region with high prevalence than in the region with low prevalence (*F* = 10.39, *p* < 0.001). Scores for stress associated with COVID-19 were significantly higher in participants in the high- and intermediate-prevalence regions than in the low-prevalence region (*F* = 6.03, *p* = 0.002). Specifically, the stress caused by economic difficulties and restrictions in daily activities was higher in the high- and intermediate-prevalence groups than in the low-prevalence group (*p* < 0.001). The people in the high-prevalence region showed significantly greater stress from anger toward people and society than those in the low-prevalence region (*F* = 5.68, *p* = 0.003). PSS scores were significantly higher in people in the high- and intermediate-prevalence regions than in the low-prevalence region (*F* = 3.45, *p* = 0.032). The scores on the PHQ-9 and the GAD-7 scale did not differ significantly among the regions.

**Table 5:** Comparisons of psychosocial distress in areas with different COVID-19 prevalence.

**Characteristics**	**Incidence of COVID-19**						
	**Total (*n* = 1,500)**	**High^**a**^ (*n* = 500)**	**Intermediate^**b**^ (*n* = 500)**	**Low^**c**^ (*n* = 500)**			
	***N* (%) or M ± SD**	***N* (%) or M ± SD**	***N* (%) or M ± SD**	***N* (%) or M ± SD**	***χ^2^ or F***	***P-value***	**Post-test (Scheffe)**
Psychosocial changes associated with COVID-19							
Fear of COVID-19 infection	3.8 ± 0.8	3.9 ± 0.8	3.8 ± 0.8	3.8 ± 0.8	2.35	0.095	
Difficulty in outside activities	4.2 ± 0.6	4.3 ± 0.6	4.3 ± 0.6	4.1 ± 0.7	12.80	<0.001	a, b > c
Disturbance in eating and sleeping	2.8 ± 0.9	3.0 ± 1.0	2.8 ± 0.9	2.7 ± 0.9	10.70	<0.001	a > c
Stigma of COVID-19	2.9 ± 0.8	2.9 ± 0.8	2.9 ± 0.8	2.9 ± 0.9	0.20	0.817	
Fear of blame for COVID-19 infection	3.6 ± 1.0	3.6 ± 1.1	3.6 ± 1.0	3.6 ± 1.0	0.22	0.806	
Stress associated with COVID-19	3.0 ± 0.8	3.1 ± 0.8	3.1 ± 0.8	2.9 ± 0.8	6.03	0.002	a, b > c
Difficult to obtain masks or hand cleaners	3.0 ± 1.2	2.9 ± 1.1	3.0 ± 1.2	2.9 ± 1.2	0.67	0.510	
Difficult to obtain daily necessities such as food or toilet paper	2.2 ± 1.1	2.3 ± 1.0	2.2 ± 1.1	2.2 ± 1.0	0.68	0.509	
Economic stress	3.5 ± 1.2	3.6 ± 1.1	3.6 ± 1.2	3.3 ± 1.1	8.84	<0.001	a, b > c
Family support stress	3.0 ± 1.2	3.0 ± 1.2	2.9 ± 1.2	2.9 ± 1.2	1.07	0.343	
Stress from daily activity changes	3.7 ± 1.0	3.8 ± 1.0	3.8 ± 1.0	3.5 ± 1.0	9.27	<0.001	a, b > c
Anger toward people and society	2.9 ± 1.1	3.0 ± 1.1	2.9 ± 1.1	2.8 ± 1.1	5.68	0.003	a > c
Perceived stress scale	19.0 ± 4.8	19.2 ± 4.9	19.2 ± 5.0	18.5 ± 4.6	3.45	0.032	a, b > c
Patient health questionare-9	5.9 ± 5.2	5.9 ± 5.1	6.3 ± 5.4	5.5 ± 5.1	2.82	0.060	
Generalized anxiety disorder-7	4.3 ± 4.5	4.4 ± 4.4	4.5 ± 4.6	3.9 ± 4.5	2.35	0.096	

a Daegu,

b Capital area,

c *Gwangju*.

## Discussion

This study revealed significant differences in psychosocial behavioral patterns and stress related to COVID-19 and general perceived stress levels according to the local prevalence of COVID-19. Disturbances in eating and sleeping and difficulty in outside activities were greater in the high-prevalence region. In addition, distress associated with economic problems, restriction of daily activities, and anger toward society were greater in people in prevalent areas. COVID-19 is not only a medical problem but also a social disaster. Because the levels of social distancing and economic stagnation might differ according to the regional prevalence of COVID-19, the psychosocial burden of COVID-19 might also differ by region. Findings of a greater distress level in the high-prevalence region in this study suggest a strong need for psychosocial interventions.

A questionnaire to evaluate psychosocial experiences and distress associated with the COVID-19 epidemic was validated in this study. The study demonstrated that social issues such as economic problems and social activity as well as psychophysiological problems such as anger and sleep difficulties were closely associated with the epidemic of COVID-19. Therefore, comprehensive investigation is required to evaluate the effects of the COVID-19 pandemic. The questionnaire used in this study could be a good tool for further research on this issue.

Although people in the high-prevalence area showed significant differences from those in the low-prevalence area in several subfactors, no significant differences existed between those in high-prevalence and intermediate-prevalence areas. This finding suggests that the threshold of COVID-19 prevalence causing distress in a community may not be very high. Although general and COVID-19-specific stress levels differed significantly according to regional prevalence. the overall level of anxiety and depression was not significantly different between regions. Factors other than the COVID-19 epidemic may contribute to depression and anxiety. However, a follow-up study is required to investigate the impacts of stress from the long persistence of the COVID-19 pandemic on general anxiety and depression. Fear of infection and stigma related to COVID-19 were not significantly different among the regions. The rapid transmission of COVID-19 and widespread media reporting may provoke fear of infection regardless of the true local COVID-19 prevalence.

According to recent studies, the occurrence of COVID-19 affects the psychosocial well-being of the public. More than half of respondents rated the psychological impact of the outbreak as moderate or severe (20), and about one-third of respondents experienced psychological distress (21). In addition, about 1/5 of the 7,236 participants suffered from depressive symptoms and sleep disturbances (22). In particular, the psychological distress scores were highest in central China (including Hubei, the center of the epidemic), which had the most outbreaks (21). As in the latter studies, from China, people living in regions with high prevalence in Korea were found to have higher distress levels, including stress from economic problems and daily activity changes. In addition, anger toward other people and society was significantly higher in the high-prevalence area than in the low-prevalence area. In the high-prevalence region, social and economic activities were more strongly inhibited, and these changes might lead to anger responses and emotional stress. Experts predict that the economic difficulties brought by COVID-19 will match the global financial crisis of 2008 (23). The past economic crisis had a negative impact on the public's mental health, such as an increased suicide rate (24). Therefore, mental health strategies for the control of emotional stress, suicidality, and anger are needed in areas influenced by the COVID-19 epidemic.

In this study, Gwangju city was selected as the region with low COVID-19 occurrence. Gwangju is the region that provides the highest level of mental health services in Korea (25). Therefore, we cannot rule out the possibility that these environmental factors might attenuate the psychosocial impacts of COVID-19.

Limitations of this study were as follows. First, an online questionnaire survey was conducted on a panel of the public belonging to a research institute. An online survey was deemed preferable to a face-to-face survey due to the need for social distancing in the context of the COVID-19 outbreak. However, results may differ between face-to-face and online surveys (26) and there is potential for selection bias. Therefore, caution is needed when comparing the results of online and face-to-face surveys. Second, the psychosocial condition before COVID-19 occurrence in each region was not evaluated. Therefore, it is necessary to interpret the results with caution and to investigate further over time. Finally, in some variables where statistically significant differences were found, the magnitude of differences among groups was small. Therefore, we should be careful when interpreting clinical implication of the differences. Despite these limitations, the study indicates a need for psychosocial support in areas with high prevalence. In addition, questionnaires for distress and psychosocial experiences associated with COVID-19 were validated by confirmatory factor analysis in this study and could be used for future related research.

## Conclusion

It is necessary to understand not only the physical effects of the COVID-19 epidemic but also the psychosocial effects (27). Our study demonstrated that psychosocial distress associated with COVID-19 was closely related to the regional prevalence of the disease, while there were no significant differences in depressive and anxiety levels across the groups. Specifically, disturbances in eating and sleeping, difficulty in outside activities, economic stress, stress from daily activity changes, anger toward society, and general perceived stress levels were greater in people in regions with high or intermediate prevalence than in people in an area with low prevalence. Psychosocial interventions for distress associated with COVID-19 should be developed and prepared for people affected by this long-lasting disease outbreak.

## Data Availability Statement

The raw data supporting the conclusions of this article will be made available by the authors, without undue reservation.

## Ethics Statement

The study was approved by the Chonnam National University Hospital Institutional Review Board. Electronic informed consent was obtained from each participant prior to starting the investigation.

## Author Contributions

SWK, MK, HK, MJ, and JYL have contributed to the conception and design of the study. IHP conducted the data collection. SWK and MK were involved in the analysis and drafted the manuscript. YSK, HK, MJ, JWK, SR, JYL, JMK, and JL critically revised the draft. All authors read and approved the submitted version.

## Conflict of Interest

The authors declare that the research was conducted in the absence of any commercial or financial relationships that could be construed as a potential conflict of interest.
